# Deep Learning-Based Classification of Inherited Retinal Diseases Using Fundus Autofluorescence

**DOI:** 10.3390/jcm9103303

**Published:** 2020-10-14

**Authors:** Alexandra Miere, Thomas Le Meur, Karen Bitton, Carlotta Pallone, Oudy Semoun, Vittorio Capuano, Donato Colantuono, Kawther Taibouni, Yasmina Chenoune, Polina Astroz, Sylvain Berlemont, Eric Petit, Eric Souied

**Affiliations:** 1 Department of Ophthalmology, Centre Hospitalier Intercommunal de Créteil, 94010 Créteil, France; karenbitton04@hotmail.com (K.B.); carlotta.pallone@gmail.com (C.P.); oudysemoun@hotmail.com (O.S.); vittorio.capuano@gmail.com (V.C.); colantuono.donato88@gmail.com (D.C.); poli_astroz@hotmail.com (P.A.); eric.souied@chicreteil.fr (E.S.); 2 Laboratory of Images, Signals and Intelligent Systems (LISSI, (EA N° 3956), University Paris-Est Créteil, 94400 Vitry sur Seine, France; taibouni.k@gmail.com (K.T.); yasmina.chenoune@esme.fr (Y.C.); petit@u-pec.fr (E.P.); 3 Keen Eye Technologies SAS, 75012 Paris, France; thomas.lemeur@keeneye.tech (T.L.M.); sylvain.berlemont@keeneye.tech (S.B.); 4 ESME Sudria, 69002 Lyon, France

**Keywords:** retinal imaging, artificial intelligence, deep learning, inherited retinal diseases, fundus autofluorescence

## Abstract

*Background*. In recent years, deep learning has been increasingly applied to a vast array of ophthalmological diseases. Inherited retinal diseases (IRD) are rare genetic conditions with a distinctive phenotype on fundus autofluorescence imaging (FAF). Our purpose was to automatically classify different IRDs by means of FAF images using a deep learning algorithm. *Methods.* In this study, FAF images of patients with retinitis pigmentosa (RP), Best disease (BD), Stargardt disease (STGD), as well as a healthy comparable group were used to train a multilayer deep convolutional neural network (CNN) to differentiate FAF images between each type of IRD and normal FAF. The CNN was trained and validated with 389 FAF images. Established augmentation techniques were used. An Adam optimizer was used for training. For subsequent testing, the built classifiers were then tested with 94 untrained FAF images. *Results*. For the inherited retinal disease classifiers, global accuracy was 0.95. The precision-recall area under the curve (PRC-AUC) averaged 0.988 for BD, 0.999 for RP, 0.996 for STGD, and 0.989 for healthy controls. *Conclusions.* This study describes the use of a deep learning-based algorithm to automatically detect and classify inherited retinal disease in FAF. Hereby, the created classifiers showed excellent results. With further developments, this model may be a diagnostic tool and may give relevant information for future therapeutic approaches.

## 1. Introduction

Inherited retinal diseases (IRDs) encompass a large, clinically and genetically heterogeneous cluster of diseases that affect around 1 in 3000 people, with a total of more than 2 million people worldwide [1]. Considering that IRDs are the most frequent inherited forms of human visual handicap, this group of diseases has a profound impact on both patients and society [2,3].

In this context, the advent of noninvasive imaging techniques has allowed for refined assessment of IRDs. Color fundus photography, fundus autofluorescence (FAF), as well as high-resolution spectral-domain optical coherence tomography have become fundamental to the diagnosis and follow up of IRDs. Of these noninvasive imaging techniques, FAF is an in vivo imaging method of the metabolic mapping of both natural and pathological fluorophores in the retina [4]. Using FAF, early stages of retinal disease as well as phenotyping (given the altered intensities of FAF imaging) are possible. The wide range of wavelengths (500–800 nm) of FAF captures lipofuscin fluorescence deposition, hence creating a topographical map of lipofuscin distribution. As lipofuscin is formed by *N*-retinyledene-*N*-retinylethanolamine (A2E), which is a metabolite from the visual cycle [5,6], this mapping of lipofuscin accumulation within the retinal pigment epithelium (RPE) provided by FAF is particularly important. Lipofuscin accumulation within the RPE is a consequence of the incomplete phagocytosis of photoreceptor outer segments by the RPE [4]. Increased lipofuscin content appears hyperautofluorescent in FAF. Moreover, it has been previously demonstrated that lipofuscin as well as its constituent A2E may exert toxic effects on normal RPE cellular processes [7,8,9]. FAF has been recently used to assess geographic atrophy (GA), patchy atrophy in pathologic myopia, as well as various monogenic IRDs, such as Best disease and Stargardt disease [5,6,10,11,12]. Interestingly, the alterations of FAF intensity are much more readily delineated on FAF images compared to color fundus photographs in IRDs such as Stargardt disease [13], Best disease [14], and retinitis pigmentosa [15]. In Stargardt disease, several phenotypes of FAF alterations can be distinguished in IRDs, from early/subclinical stages to advanced stages [12,13,14,15], proving that this type of imaging may be helpful not only for the detection of affected areas but also for the differential diagnosis and the follow up of IRDs [12]. In particular, FAF can capture the high-concentration intracellular A2E as hyperautofluorescent small lesions, corresponding to the flecks typically seen in Stargardt disease [12,13]. Moreover, atrophy, appearing in late stages of Stargardt disease, would appear hypoautofluorescent due to the disappearance of both RPE and choriocapillaris [12,13]. In retinitis pigmentosa, the most common FAF phenotype is the hyperautofluorescent Robson–Holder parafoveal ring. Interestingly, the Robson–Holder ring is not visualized on color fundus photography [12,15]. In Best disease, several progression stages are reflected by FAF imaging, with a progression from increased autofluorescence to atrophy of the photoreceptors leading to decrease in the hyperautofluorescence and finally to damage to the RPE. [6,12,14].

Nevertheless, IRDs are relatively rare, with an estimated prevalence of 1 in 16,500 to 1 in 21,000 for Best disease [16], 1 in 8000 to 1 in 10,000 for Stargardt disease [2,17], and 1 in 4000 for retinitis pigmentosa [18]. Given their rarity, these conditions are often difficult to diagnose and patients can endure a long journey involving many ophthalmologists. Over the past years, machine learning and more recently deep learning have been increasingly applied to a vast array of ophthalmological diseases from diabetic retinopathy (DR) [19] to age-related macular degeneration (AMD) [20,21] and glaucoma [22], with sensitivities and specificities above 90% [19,20,21,22,23,24]. Deep learning approaches were also in used in less frequent conditions, for instance to detect and to analyze to progression of chorioretinal atrophy [25] or to distinguish GA from Stargardt diseases using FAF imaging [26]. Deep learning is based on artificial neural networks, inspired by the biological neural networks [23]. The self-learning algorithms in the deep convolutional neural network (CNN) allow for automated detection of different structures. CNN is a class of artificial neural networks that is most commonly used for image recognition and classification. However, deep learning approaches for automated image analysis require large volumes of high-quality training data, which may be a challenging premise in a clinical setting. These high volumes of data are even more difficult to obtain in the case of IRDs due to the rareness of these genetic conditions. We describe a preliminary study to evaluate the use of deep learning for the automated classification of FAF images from a cohort of patients with Stargardt disease (STGD), Best disease (BD), and Retinitis Pigmentosa (RP).

## 2. Methods

### 2.1. Datasets

This retrospective study was conducted in accordance with the tenets of the Declaration of Helsinki. This study had the approval of the Ethics Committee of the Federation France Macula 2018-27 and was carried out in compliance with French legislation. Written consent was waived because of the retrospective nature of the study. This retrospective analysis included FAF imaging of patients from the Department of Ophthalmology of the University Paris Est Creteil who presented between April 2007 and April 2019 with three of the most frequent IRDs: Stargardt disease (STGD), Best diseases (BD), and Retinitis Pigmentosa (RP). Diagnosis of STGD, BD, and RP for the included eyes was substantiated using clinical data, mode of inheritance, multimodal imaging, electroretinogram (ERG) findings, and molecular genetic testing, when available, by two retina specialists (A.M. and K.B.). Patients with no evidence of retinal disease as determined by a retina specialist were defined as healthy controls. We used macula-centered fundus autofluorescence retinal images from the Department of Ophthalmology of Créteil, France. FAF images had been obtained in the Ophthalmology outpatient clinic in the Department of Ophthalmology in Créteil between April 2007 and April 2019, using Spectralis HRA + OCT (Heidelberg Eye Explorer, Version 1.10.4.0, Heidelberg Engineering, Heidelberg, Germany). High-resolution (1536 × 1536 pixels), 30 × 30, and 55 × 55 degree-field-of-view images centered on the fovea with a minimum average of 30 frames were captured. All images were deidentified, and all personal data (e.g., patient name, birth date, and study date) were removed. FAF images were cropped to a size of 768 × 768 pixels with the fovea at the center. Images were labeled as either normal, STGD, BD, or RP by two retina specialists (A.M. and K.B.). A four-class classification system (normal, STGD, BD, and RP) was implemented. The images were partitioned in three sets: the training set (70% of the images), the validation set (10% of the images), and the test set (20% of the images). Assignment of the images towards the training, the validation, and testing set was performed randomly. Images were separated at eye-level to strictly separate the training data from the validation and test data to prevent intra-eye correlations. Images that were used for training the deep learning classifier were not used to test it.

### 2.2. Development of a Deep Learning Classifier

**Figure 1:**
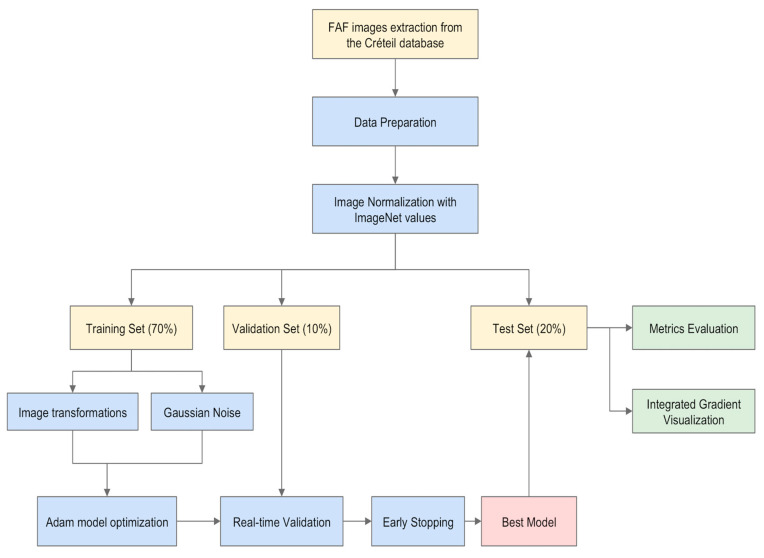
Illustration of the development of a deep learning classifier: fundus autofluorescence images of Stargardt disease, retinitis pigmentosa, and Best vitelliform macular dystrophy as well as of healthy controls were extracted from the Créteil database. After data preparation, transfer learning from the ImageNet dataset (http://www.image-net.org/) was used. The images were randomly partitioned in three sets: the training set (70% of the images), the validation set (10% of the images), and the test set (20% of the images). Data augmentation was performed on the training set to increase the original dataset and to reduce overfitting of the final model. The images were normalized using the mean and standard deviation of the ImageNet dataset to match the model initialization. The model was optimized using Adam Optimization Algorithm during 5000 iterations. The model was then evaluated with the test set of 94 images. The output of the model was the metric evaluation of the performance of the model (accuracy, sensitivity, and specificity) and integrated gradient visualization.

For this study, the deep learning framework TensorFlow™ (Google Inc., Mountain View, CA, USA) was used. We used ResNet 101 (Microsoft ResNet; Microsoft Research Asia, Beijing, China) to perform the classification task [27]. This deep CNN is widely used for image classification. ResNet 101 has the advantage of introducing residual connections to increase the network depth without negative outcomes and to thus improves classification results [27,28]. Transfer learning from the ImageNet dataset (http://www.image-net.org/) was used to provide base knowledge to the CNN before fine-tuning it. To fit our task, we reduced the number of output neurons in the last fully connected layer to four. Moreover, we fixed the first ResNet 101 block during the training process to keep low-level features learned on ImageNet and to speed up the training. Data augmentation was used to increase the original dataset and to reduce overfitting of the final model. This was achieved through a combination of image translation, cropping, and rotation. Moreover, Gaussian noise augmentation was used to mimic low-quality noisy images. The images were normalized using the mean and standard deviation of the ImageNet dataset to match the model initialization. The model was optimized using Adam Optimization Algorithm during 5000 iterations [29]. The model was then evaluated with the test set of 94 images. By using integrated gradients, attribution maps were generated, allowing to assess the impact of each pixel in the classification and showing on which areas the model relies to perform the classification [30]. The method is summarized in Figure 1.

Performance was evaluated through a comparison of the CNN output to the ground truth, set by clinical diagnosis by expert readers. Three metrics were used for this purpose: accuracy, sensitivity, and specificity. Confusion matrices, area under (AUC) receiver operating characteristics (ROC), and precision-recall (PRC) curves were generated. The deep learning model’s confidence was assessed using softmax regression on the test set. In addition, multiple Kernel density estimation (KDE), a nonparametric probability density estimation, was also generated to compare the model’s confidence throughout the four classes.

## 3. Results

The data used to train, validate, and test the algorithm were composed of 73 FAF images from participants with a normal retina and 410 FAF images from participants with IRDs: 125 FAF images from patients with STGD, 160 FAF images from patients with RP, and 125 FAF images from patients with BD. Of these, 389 FAF images were used for training and validation and the remaining 94 FAF images (23 STGD, 32 RP, 25 BD, and 14 healthy controls) were used for testing. For STGD, the ROC-AUC was 0.998, the PRC-AUC was 0.986, the sensitivity for STGD FAF image classification was 0.96, and the specificity was 1. For RP, the ROC-AUC was 0.999, the PRC AUC was 0.999, the sensitivity was 1, and the specificity averaged 0.97. For BD, the ROC-AUC was 0.995, the PRC AUC was 0.988, the sensitivity averaged 0.92, and the specificity averaged 0.97. For healthy controls, the ROC-AUC was 0.998, the PRC-AUC was 0.989, the sensitivity for normal FAF image classification was 0.86, and the specificity averaged 0.99. The overall accuracy for the classification was 0.95.

**Figure 2:**
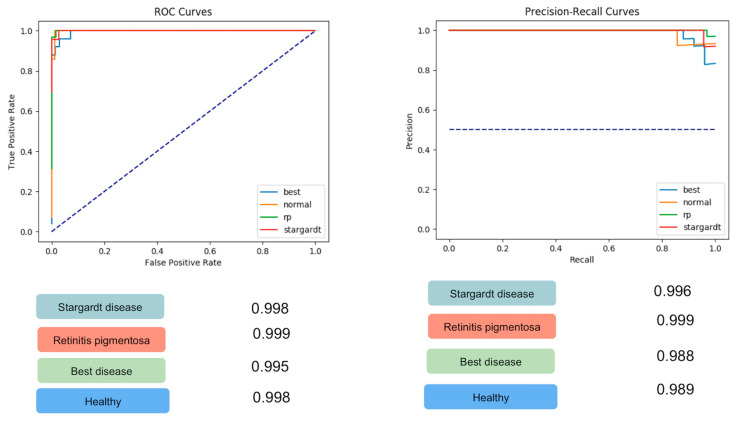
Receiver operating characteristics (ROC) and precision-recall (PRC) area under the curve (AUC) for the 4 classes: Stargardt disease (STGD), Retinitis pigmentosa (RP), Best vitelliform macular dystrophy (BD), and healthy controls. For STGD, the ROC-AUC (left panel) was 0.998 and the PRC-AUC (right panel) was 0.986. For RP, the ROC-AUC (left panel) was 0.999 and the PRC AUC (right panel) was 0.999. For BD, the ROC-AUC (left panel) was 0.995 and the PRC AUC (right panel) was 0.988. For healthy controls, the ROC-AUC (left panel) was 0.998 and the PRC-AUC (right panel) was 0.989.

These results are summarized in Table 1 and Table 2 and Figure 2.

**Figure 3:**
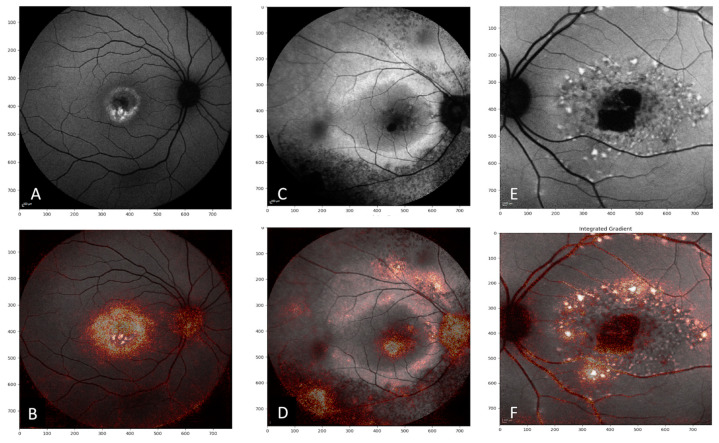
Fundus autofluorescence (FAF) images (upper panels) of correct attribution with integrated gradient visualization (lower panels): (**A**) FAF image of Best vitelliform macular dystrophy, correctly classified by the deep learning model; (**B**) integrated gradient visualization reflects distinctive features (in this case, the presence of hyperautofluorescent vitelliform deposit) of the input image; (**C**). FAF image of retinitis pigmentosa, correctly classified by the deep learning model; (**D**). integrated gradient visualization reflects distinctive features (in this case, the presence of hyperautofluorescent ring) of the input image; (**E**) FAF image of Stargardt disease, correctly classified by the deep learning model; and (**F**) integrated gradient visualization reflects distinctive features (in this case, the presence of hyperautofluorescent flecks and centra atrophy) of the input image.

**Figure 4:**
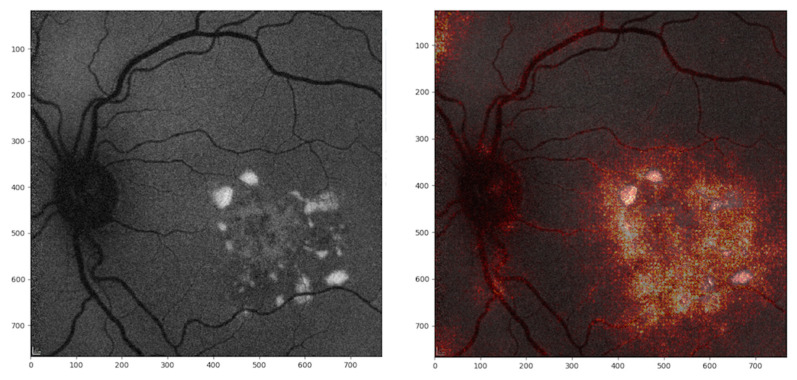
Fundus autofluorescence (FAF) image (left panel) with integrated gradient visualization of incorrect attribution (right panel) in a case of Stargardt disease: the hyperautofluorescent flecks present in the central macular region have determined a predicted diagnosis of Best disease by the deep learning model.

Figure 3 illustrates integrated gradient visualization for correct attributions, while Figure 4 illustrates integrated gradient visualization for incorrect attributions.

**Figure 5:**
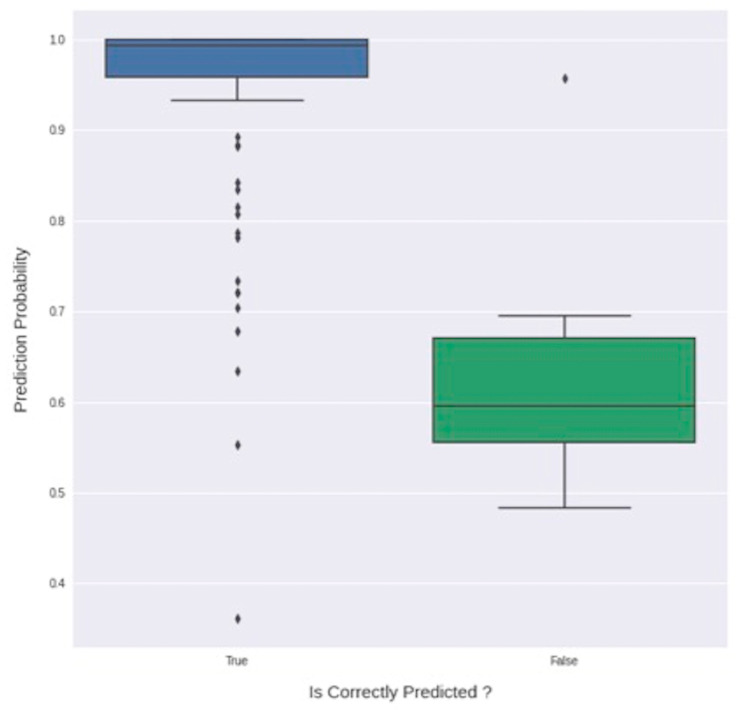
Distribution of softmax probabilities for correct and erroneous predictions on the test set: note that the average confidence probability for correctly predicted test images was 0.943 (median 0.993) (blue box), whereas the average confidence probability for erroneously predicted elements was 0.645 (median 0.595) (green box).

In order to assess model uncertainty, softmax regression was employed on the test set. The average confidence probability for correctly predicted test images was 0.943 (median 0.993), whereas the average confidence probability for erroneously predicted elements was 0.645 (median 0.595) (Figure 5). 

**Figure 6:**
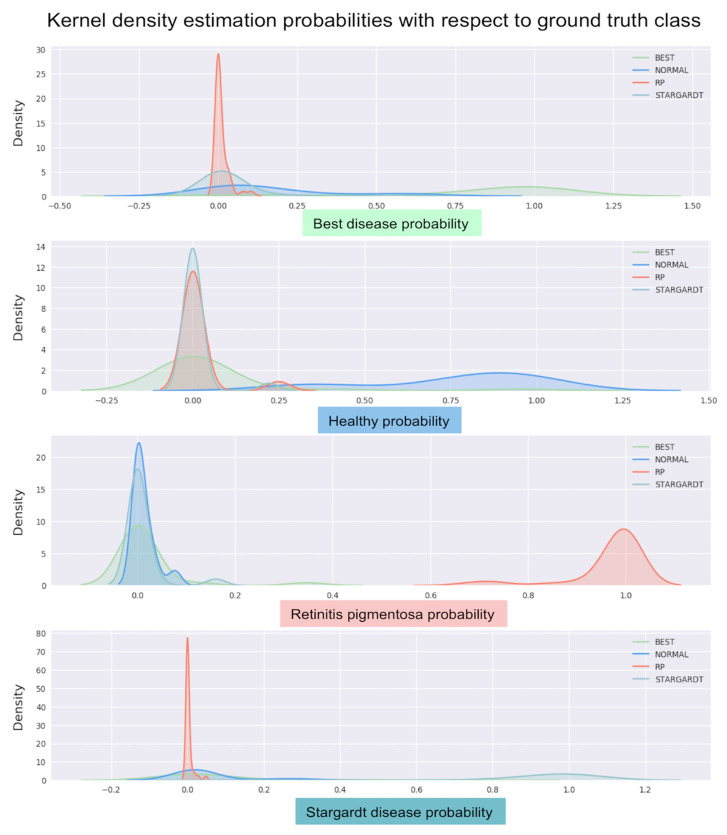
Kernel density estimation (KDE) graphs showing the estimated probability for each of the four classes: upper panel, Best disease KDE; second panel, healthy control KDE; third panel, retinitis pigmentosa KDE; and fourth panel, Stargardt disease KDE. The deep learning model generates a probability for the affiliation of each image of the test dataset to one of the four classes. The images are then compared to each ground truth class to which they were originally assigned, leading to four different KDE graphs. A peak towards 1 corresponds to FAF images for which the probability of the predicted class coincides with the tested ground truth class, while a peak towards 0 corresponds to FAF images for which the probability for the tested ground truth class is low and is, therefore, not likely to correspond to the studied ground truth class.

Moreover, multiple KDE graphs show the highest estimated probability for each of the four classes (Figure 6).

The model was then trained separately only with 30 × 30 degree-field-of-view and 55 × 55 degree-field-of-view FAF images of the four classes, obtaining a classification accuracy of 0.94 for 30 × 30 degree-field-of-view FAF images and of 0.94 for 55 × 55 degree-field-of-view FAF images. The confusion matrices corresponding to these trainings are shown in Table 3.

## 4. Discussion

In this study, we demonstrated the feasibility of automated classification of several IRDs using FAF images, employing a convolutional neural network. Our study showed high sensitivity and specificity, with an overall accuracy of 0.95. Fundus autofluorescence imaging, providing a metabolic mapping of the retina, provides crucial information for the diagnosis of IRDs and typical phenotypes in each of the IRDs included in this study. Stargardt disease is produced by a mutation in the ABCA4 gene. The ABCA4 gene product is an ATP-binding cassette transporter that transports all-trans-retinol produced in a light-exposed photoreceptor outer segment to the extracellular space [31,32]. As a result of the mutation, A2E (N-retinylidene-N-retinylethanolamine) accumulates within the outer segment of photoreceptors, subsequently phagocytosed by RPE cells. As an in vivo metabolic mapping of the retina, FAF can visualize the high-concentration intracellular A2E as hyperautofluorescent lesions, corresponding to the flecks typically seen in Stargardt disease [12,13]. In late stages of Stargardt disease, hypoautofluorescent atrophy is visible on FAF due to the disappearance of both RPE and choriocapillaris [12,13,32].

The most frequent FAF phenotype in retinitis pigmentosa is the hyperautofluorescent Robson–Holder parafoveal [12,15], which is not visualized on color fundus photography. The parafoveal hyperautofluorescent ring may be a result of rod system dysfunction, according to the distribution of rod photoreceptors and to the low density of cones outside the foveal area [12]. In the rest of the cases, FAF in retinitis pigmentosa displays abnormal central hyperautofluorescence extending centrifugally from the fovea in 18% of cases and neither pattern in 24% of cases [12,33].

Best vitelliform macular dystrophy is caused by mutations in the BEST1 gene located on chromosome 11q13, which encodes bestrophin-1, a protein localized to the basolateral surface of the RPE. Spaide et al. hypothesized that the central accumulation of a well-demarcated hyperautofluorescent vitelliform deposit is subsequent to the inadequate removal of subretinal fluid, leading to physical separation of the photoreceptors from the RPE, progressively resulting in the accumulation of lipofuscin at the outer side of the neurosensory retina (due to shedding of the outer segment discs that cannot be phagocyted by the RPE cells) [6]. Several progression stages of BD are reflected by FAF imaging, from initial increased autofluorescence to late-stage atrophy of the photoreceptors [6,12,14]. Fundus autofluorescence is therefore an important tool for the phenotypic characterization of retinal dystrophies.

Furthermore, by using integrated gradient visualization, we were able to ascertain the impact of each pixel in the classification and to visualize areas that the model relies on to predict one class or the other. Interestingly, by using integrated gradient visualization, the regions of interest for CNN corresponded to the areas of interest described above, i.e., increased autofluorescence either parafoveal, such as in the Robson–Holder ring, or focal, in flecks or in vitelliform deposits. When well-circumscribed, hypoautofluorescent atrophy was present, integrated gradient visualization allowed to demonstrate that this feature was also taken into account. These findings are illustrated in Figure 3. However, no reference databases to classify consistently the normal and pathological FAF phenotypes are available. 

To date, the use of both deep learning classification for IRDs and of FAF imaging for deep learning purposes have been scarce in the literature. Concerning the automated classification of IRDs, there has been one study by Fujinami-Yokokawa et al. [34], using OCT and a commercially available deep learning platform (Inception-v3 CNN) [35]. The authors reported a mean overall test accuracy of 0.909. However, in their study, three different OCT devices were used and the total number of OCT macular images was 178. Moreover, the authors performed four repeated tests, with a significant increase in test accuracy, which may suggest overfitting of their model. Recently, Shah et al. demonstrated that it is possible to use deep learning classification models to differentiate between normal OCT images and STGD OCT images and to distinguish the severity of STGD from OCT images. The authors used on a small dataset a pretrained model (VGG19) and a new classification model, obtaining an accuracy of 0.996, a sensitivity of 99.8%, and a specificity 98.0% for the pretrained model and an accuracy of 0.979, a sensitivity 97.9%, and a specificity 98.0% with the new classification model [36]. The high accuracy, sensitivity, and specificity are consistent with our results (Table 1).

FAF imaging has been used in a deep learning-based algorithm to automatically detect and classify GA by Treder et al. [21] as well as to detect chorioretinal atrophy by Ometto et al. [26] and to distinguish GA from Stargardt disease by Wang et al. [27]. In the study performed by Treder et al., two classifiers were built to differentiate between GA and healthy-eye FAF images and between GA and a group named other retinal diseases (ORD), with a training and validation set of 200 GA FAF images, 200 healthy-eye FAF images, and 200 FAF images of ORD. The test set consisted of 60 untrained FAF images in each case (GA 30, healthy 30, or ORD 30). For the GA classifiers, their model achieved a training accuracy of 99/98 and a validation accuracy of 96/91. Wang et al. used 320 FAF images from normal subjects, 320 FAF images with GA, and 100 with Stargardt disease in atrophic stage, obtaining a high screening accuracy with 0.98 for GA and 0.95 for atrophic Stargardt disease [27]. The excellent results confirm that automated classification with a deep learning classifier is possible in GA using FAF images.

Considerable efforts continue to be made to develop automated image analysis systems for the precise detection of disease in several medical specialties. In recent years, the use of CNNs has become increasingly popular for feature learning and object classification. Following the ImageNet Large Scale Visual Recognition Challenge, Russakovsky and collaborators demonstrated that the object classification capabilities of CNN architectures can surpass those of humans [37]. While the applications of artificial intelligence have mainly focused on diabetic retinopathy and age-related macular degeneration or glaucoma [19,20,21,22,23], IRDs would make an interesting candidate due to the typical, symmetrical phenotype of these disorders.

Our study has several limitations, one of which is the use of a small dataset. Moreover, our deep learning classifier was trained to only distinguish between three of the less rare IRDs, for which we have sufficient training data. Moreover, eye-level partitioning of the dataset and the use of a training/validation/test split are other limitations due to the sample variability and to the fact that, when training on different images, the model might not perform as well. Lack of molecular genetic testing for a part of the included eyes is another limitation. Due to the vast spectrum and genotypic and phenotypic variability of IRDs, it is difficult to assess how such a classifier would perform in a clinical setting. Moreover, image noise and the presence of interindividual and intraindividual variability in terms of media opacities, lipofuscin content, and genetic expression impact FAF imaging and may become a significant challenge.

Confidence estimation allows for quantifying model uncertainty. This is of the utmost importance when the deep learning model has to make predictions in a clinical setting, possibly on out of distribution data (therefore different from the distribution of the data on which the model was trained) resulting in variations in accuracy. Interestingly, in our series, the average confidence probability for when the predicted class for FAF images was correct was 0.943 (median 0.993), whereas the average confidence probability for the erroneously predicted FAF images was 0.645 (median 0.595). This analysis shows that, when correctly classifying images, the deep learning model is more confident than when it classifies images incorrectly (Figure 5). Furthermore, KDE graphs (Figure 6) offer complementary information on the model’s confidence in correctly predicting the four classes.

Moreover, our limited dataset made further classifications according to the disease stage impossible. Nevertheless, given that IRDs are orphan diseases, large datasets would only be available through multi-institutional collaborations. With further developments, this model may be a diagnostic tool and may give relevant information for future therapeutic approaches.

## Figures and Tables

**Table 1:** Area under the curve (AUC) for receiver operating characteristics (ROC) and precision-recall (PRC) curves and sensitivity and specificity of the deep learning classifier for inherited retinal diseases (IRDs) fundus autofluorescence (FAF) images.

Class	ROC-AUC	PRC-AUC	Sensitivity	Specificity
Stargardt disease FAF	0.998	0.986	0.96	1
Retinitis pigmentosa FAF	0.999	0.999	1	0.97
Best disease FAF	0.995	0.988	0.92	0.97
Healthy controls FAF	0.998	0.989	0.86	0.99

**Table 2:** Confusion matrix of the deep learning classifier test dataset on a total of 94 fundus autofluorescence (FAF) images of inherited retinal disease (23 Stargardt disease, 32 retinitis pigmentosa, and 25 Best disease images) and healthy controls (14 images).

Ground Truth Class	Predicted Class			
	Stargardt Disease	Retinitis Pigmentosa	Best Disease	Healthy Control
**Stargardt Disease**	22	0	1	0
**Retinitis Pigmentosa**	0	32	0	0
**Best Disease**	0	1	23	1
**Healthy Control**	0	1	1	12

**Table 3:** Confusion matrix of the deep learning classifier test dataset for 30 × 30 degree-field-of-view (test dataset = 66 images) and 55 × 55 degree-field-of-view (test dataset = 32 images) fundus autofluorescence (FAF) images.

**30 × 30 Degree-Field-of-View**				
**Ground Truth Class**	**Predicted Class**			
	**Stargardt Disease**	**Retinitis Pigmentosa**	**Best Disease**	**Healthy Control**
**Stargardt Disease**	10	0	1	0
**Retinitis pigmentosa**	0	24	0	0
**Best disease**	0	0	17	0
**Healthy control**	0	0	2	11
**55 × 55 Degree-Field-of-View**				
**Ground Truth Class**	**Predicted Class**			
	**Stargardt Disease**	**Retinitis Pigmentosa**	**Best Disease**	**Healthy Control**
**Stargardt disease**	9	0	0	0
**Retinitis pigmentosa**	0	17	0	0
**Best disease**	2	0	4	0
**Healthy control**	0	0	0	0
